# Mesenchymal stromal cells derived from acute myeloid leukemia bone marrow exhibit aberrant cytogenetics and cytokine elaboration

**DOI:** 10.1038/bcj.2015.17

**Published:** 2015-04-10

**Authors:** J C Huang, S K Basu, X Zhao, S Chien, M Fang, V G Oehler, F R Appelbaum, P S Becker

**Affiliations:** 1 Division of Hematology, Institute for Stem Cell and Regenerative Medicine, Seattle, WA, USA; 2 Division of Gerontology & Geriatric Medicine, University of Washington, Seattle, WA, USA; 3 Section of Hematology & Oncology, West Virginia University School of Medicine, Morgantown, WV, USA; 4 Division of Clinical Research, Fred Hutchinson Cancer Research Center, Seattle, WA, USA; 5 Department of Pathology and Laboratory Medicine, University of Washington, Seattle Cancer Care Alliance, Seattle, WA, USA; 6 Division of Medical Oncology, Department of Medicine, University of Washington, Seattle, WA, USA

## Abstract

Bone marrow-derived mesenchymal stromal cells (BM-MSCs) play a fundamental role in the BM microenvironment (BME) and abnormalities of these cells may contribute to acute myeloid leukemia (AML) pathogenesis. The aim of the study was to characterize the cytokine and gene expression profile, immunophenotype and cytogenetics of BM-MSCs from AML patients compared to normal BM-MSCs from healthy donors. AML BM-MSCs showed decreased monocyte chemoattractant protein-1 levels compared to normal BM-MSCs. AML BM-MSCs expressed similar β1 integrin, CD44, CD73, CD90 and E-cadherin compared to normal BM-MSCs. Cytogenetic analysis revealed chromosomal aberrations in AML BM-MSCs, some overlapping with and others distinct from their corresponding AML blasts. No significant difference in gene expression was detected between AML BM-MSCs compared to normal BM-MSCs; however, comparing the differences between AML and MSCs from AML patients with the differences between normal hematopoietic cells and normal MSCs by Ingenuity pathway analysis showed key distinctions of the AML setting: (1) upstream gene regulation by transforming growth factor beta 1, tumor necrosis factor, tissue transglutaminase 2, CCAAT/enhancer binding protein alpha and SWItch/Sucrose NonFermentable related, matrix associated, actin dependent regulator of chromatin, subfamily a, member 4; (2) integrin and interleukin 8 signaling as overrepresented canonical pathways; and (3) upregulation of transcription factors FBJ murine osteosarcoma viral oncogene homolog and v-myb avian myeloblastosis viral oncogene homolog. Thus, phenotypic abnormalities of AML BM-MSCs highlight a dysfunctional BME that may impact AML survival and proliferation.

## Introduction

Acute myeloid leukemia (AML) is a heterogeneous disorder that arises in the bone marrow microenvironment (BME) under the influence of mesenchymal stromal cells (MSCs), endothelial cells, osteocytes, pericytes, adipocytes, monocytes, fibroblasts and their secreted extracellular glycosaminoglycans, chemokines and cytokines. The BME plays a fundamental role in the growth, proliferation and survival of AML cells. Leukemia cell growth in the BME also disrupts normal hematopoiesis.^1^ Reciprocal interactions between the BME and AML cells through paracrine and autocrine signaling molecules along with cell–cell and cell–matrix adhesion promote leukemia cell quiescence, activation of pro-survival and anti-apoptotic pathways, chemotherapy resistance and minimal residual disease.^2, 3, 4^ Furthermore, disruption of adhesion-mediated interactions with malignant cells can overcome environment-mediated chemotherapy resistance.^5, 6, 7^ Accumulating evidence has shown that the cancer microenvironment directly contributes to the pathogenesis, treatment resistance or relapse of various malignancies. For example, growth factors from stromal cell lines confer resistance to targeted therapies and chemotherapy in melanoma, glioblastoma and colorectal cancer cell lines in a co-culture system.^8^ Chemotherapy-induced damage of benign prostatic stromal cells resulted in diminished chemotherapy effect and promoted prostate cancer cell survival and progression.^9^ In breast cancer, tumor cell gene expression changed when co-cultured on different tissue microenvironments.^10^ In a study of surgically resected hepatocellular carcinoma, the gene expression profile of the non-malignant peri-tumoral tissue in the resection specimen could predict disease relapse, whereas the expression profile of the malignant cells themselves did not.^11^ These findings suggest that alteration in the surrounding stromal tissue independently contributed to disease behavior. Work in mouse models have provided evidence that alterations in the BME can contribute to the evolution of hematologic malignancy: for example, knockout of the RNase III endonuclease *Dicer1* specifically in mesenchymal osteoprogenitor cells resulted in myelodysplasia and emergence of AML despite maintaining genetic integrity in the hematopoietic lineage.^12^ Another study showed that an activating mutation of β-catenin in osteoblasts induced AML through Notch signaling.^13^ Considering these reports of stroma-microenvironment aberrations specifically influencing the evolution of malignant conditions, we hypothesized that the BM-derived MSCs (BM-MSCs) from AML patients would exhibit distinct genotypic and phenotypic differences compared to BM-MSCs from normal healthy donors.

The central component of the BME are MSCs, which are capable of giving rise to different cell lineages, such as osteoblasts, adipocytes and chondroblasts.^14^ Cytogenetic abnormalities have been reported in BM-MSCs from patients with myelodysplastic syndrome (MDS) and AML, which were distinct from leukemic blasts and may be associated with inferior outcomes.^15^ A distinctive gene expression profile of MSCs from pediatric cases of MDS and AML was found compared with healthy donors.^16^ To gain further insight into the characteristics of MSCs in AML, we aimed to characterize the cytokine and gene expression profile, immunophenotype and cytogenetics of primary BM-MSCs isolated from AML patients in comparison to healthy donors.

## Subjects and methods

### Human studies

The study was conducted with written informed consent in accordance with the Declaration of Helsinki and under the guidelines of the University of Washington/Fred Hutchinson Cancer Research Center (FHCRC) Cancer Consortium Institutional Review Board, which approved the study.

### Primary BM-MSC culture

MSC cultures were successfully derived from BM aspirates of 22 AML patients and 5 healthy donors, either from fresh or cryopreserved samples. Mononuclear cells (MNCs) were isolated from BM samples using lymphocyte separation medium (Media Tech Inc., Manassas, VA, USA). Approximately, 2 to 5 million fresh or cryopreserved BM cells were cultured overnight in Iscove's Modified Dulbecco's Media (Sigma-Aldrich, St Louis, MO, USA), 15% heat-inactivated fetal bovine serum, 15% horse serum, 1% penicillin–streptomycin (media and supplements from Invitrogen, Carlsbad, CA, USA), then the media was replaced (removing the non-adherent cells) over the adherent cells by non-hematopoietic expansion media (NHEM, Miltenyi, Auburn, CA, USA) in 5% oxygen within a hypoxic chamber to more closely approximate the sinusoidal regions of the BM. Cells were also cultured in normal 21% oxygen conditions for comparison of cytokine production at different oxygen concentrations. After 48 h, non-adherent cells were removed from the culture by washing with phosphate-buffered solution. Over a period of 1–2 weeks, adherent, spindle-shaped cells were derived and subcultured weekly by trypsinization for ~10–15 passages. The MSCs were collected for analysis after the third to fourth passages. Similar cultures were derived from normal BM from healthy donors and normal homo sapiens (HS)-5 and HS-27a stromal cell lines (kindly provided by Dr Beverly Torok-Storb, FHCRC) were also used for comparison.

### Immunophenotype of mesenchymal stromal cells by flow cytometry

Flow cytometry was performed on single stromal cell suspensions that were stained with the following anti-human monoclonal antibodies: allophycocyanin-conjugated antibody against CD90, CD34 or CD45, fluorescein isothiocyanate-conjugated antibody against CD324 or CD146, R-phycoerythrin-conjugated antibody against CD14, CD29, CD44 or CD73, and PerCP Cy5-5-conjugated antibody against CD105 to confirm their mesenchymal origin.

### Cytokine production assays

Conditioned media was collected from adherent, subconfluent MSC cultures (5 and 21% oxygen) after cell components were removed by centrifugation. The Luminex method (Luminex, Austin, TX, USA) was used for detection of the following cytokines: granulocyte colony-stimulating factor (G-CSF), granulocyte-monocyte CSF (GM-CSF), macrophage CSF, stem cell factor, interleukin 6 (IL-6), IL-12, monocyte chemoattractant protein-1 (MCP-1) and tumor necrosis factor alpha (TNF-α). Enzyme-linked immunosorbent assay was used for detection of stromal-derived factor-1. All assays were performed at the FHCRC Immunoassays Core Facility according to manufacturer's instructions.

### Cytogenetics and fluorescence *in situ* hybridization

Cells actively growing in logarithmic phase were incubated with 0.1 μg/ml colcemid for 60 min at 37 °C to arrest cells in metaphase. Cells were then treated with the pre-warmed hypotonic solution (0.075 M KCl) for 30 min before fixation in methanol and glacial acetic acid (2.5:1 ratio). Fixed cells were dropped on glass slides, treated with 0.025% trypsin in 0.9% NaCl and stained with 1:4 diluted Wright's stain (pH 6.8) for Giemsa-Trypsin-Wright banding. Metaphases were analyzed under a microscope at a magnification of × 1250 for chromosome count and structural integrity. At least 20 metaphases were analyzed for each sample. Karyotypes were written according to current International System for Human Cytogenetic Nomenclature (ISCN 2013).^17^ Fluorescence *in situ* hybridization was performed on one sample using a centromere region of chromosome 8p11.1-q111.1 probe (Abbott Molecular, Des Plaines, IL, USA). Slides were enumerated using AxioImager Z1/2 microscopes (Zeiss, Oberkochen, Germany) on 600 interphase nuclei. Clonal results above the laboratory-established false-positive cutoff values were considered positive.

### Gene expression microarray

For all patients and cell lines, 100 ng of high-quality RNA was used. Total RNA was extracted using RNeasy mini kit (Qiagen, Valencia, CA, USA), labeled and hybridized to Illumina's HumanHT Expression BeadChip microarrays according to the manufacturer's instructions by the FHCRC Genomics Core Facility. Signal intensities were quantile normalized using the ‘lumen package' from Bioconductor (http://www.bioconductor.org/) and log-2 transformed. Significant differential gene expression was defined as |log2(ratio)|>0.585 (±1.5-fold) and false discovery rate of 5%. Probe sets retained after application of signal intensity and variance filters were used in a principal component analysis^18^ to generate a map of AML BM-MSCs, AML blasts, normal BM-MSCs, normal hematopoietic MNCs and the stromal cell lines (HS-5 and HS-27a) by projection of the data onto the first two principal components (that is, the two directions that explain the most variance in the dimensional space of standardized gene expression values). These probe sets were also used to generate a heat map (Supplementary Figure 1) that met the following conditions |log2 (ratio)|>0.585 and *P*-value <0.002. Each sample's intensity value was divided by the row-wise median (across all samples). These values were hierarchically clustered by probe.

### Ingenuity pathway analysis

GenePlus software (Enodar Biologic, Seattle, WA, USA), which uses estimating equation techniques was also used to determine differential expression between groups using analysis of variance to calculate *P*-values, *z*-scores and the number of false discoveries. Differences in gene expression between AML BM-MSCs and AML blasts were compared with the differences in gene expression between normal BM-MSCs and normal hematopoietic cells through use of Qiagen's Ingenuity pathway analysis software (IPA, Ingenuity Systems, Inc., 2014, Redwood City, CA, USA; http://www.ingenuity.com). IPA determines biologically enriched pathways and functions based on direct and indirect relationships in published literature. The *P*-value is calculated using a right-sided Fisher's exact test and measures the statistical significance of a particular function or pathway in our data with respect to the reference set defined by IPA. A key feature of IPA is the upstream regulator analysis, which identifies putative upstream regulators that are activated or inhibited in our gene expression data sets. Upstream regulators include transcription factors, cytokines, microRNAs, receptors, kinases, chemicals and drugs. Activation or inhibition is assigned based on relationships derived from the literature and various databases and the direction of expression change in the array data provided. It also identifies candidates that are statistically enriched when the direction cannot be ascertained. The *P*-value is calculated using Fisher's exact test.

### Statistics

Results in cytokine levels are the average of experiments carried out in duplicate. Comparisons in cytokine levels between different groups (AML vs healthy donors) and different oxygen conditions (5 vs 21% O_2_) were made using the Student's *t*-test and the two-sided Fisher's exact test (dichotomous variables). *P*-value <0.05 was considered statistically significant.

## Results

### Morphology of BM-MSCs derived from AML patients

The spindle-shaped cells grew adherent to the culture dish and became subconfluent or confluent by 1–2 weeks. The cells were fibroblast like in morphology with a few long processes and large nuclei (Supplementary Figure 2). The MSCs were subcultured weekly by trypsinization and could be passaged for 10–15 times.

### Immunophenotype of BM-MSCs from AML patients and healthy donors

**Figure 1:**
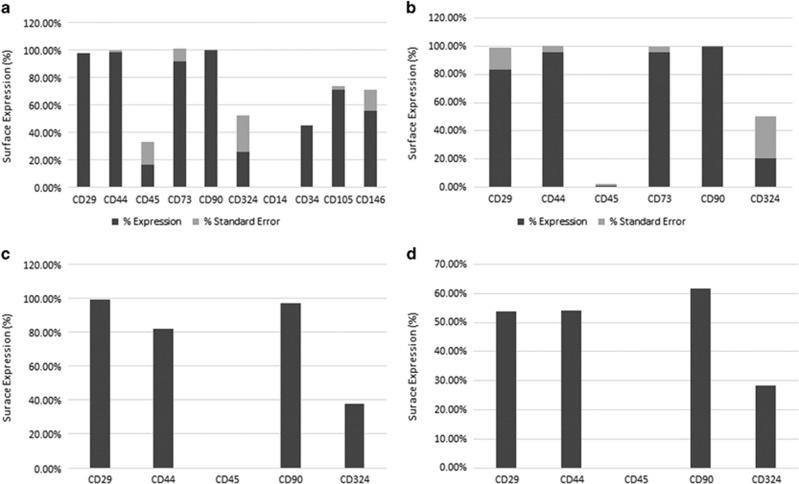
Characterization of the immunophenotype of AML BM-MSCs, normal BM-MSCs and stromal cell lines by flow cytometry reveals similar surface marker expression. (**a**) Surface marker expression of AML BM-MSCs (*n*=3). (**b**) Surface marker expression of BM-MSCs from healthy donors (*n*=3). (**c**) Surface marker expression of stromal cell line HS-5 (*n*=1). (**d**) Surface marker expression of stromal cell line HS-27a (*n*=1).

**Figure 2:**
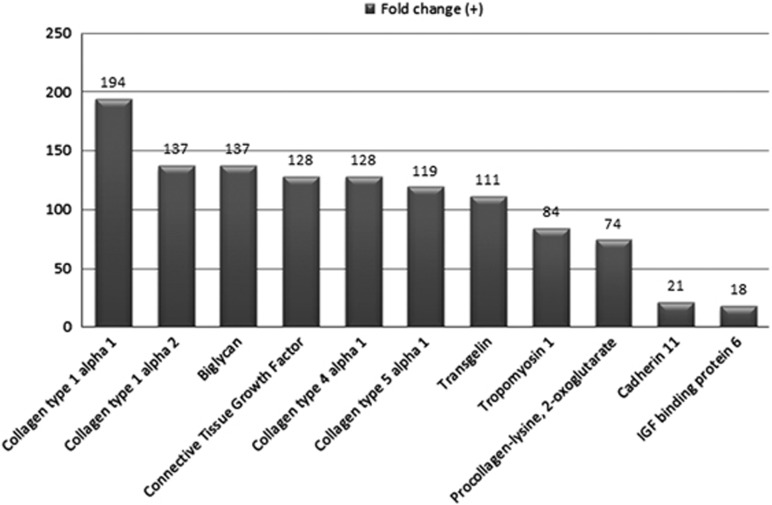
Differential gene expression between AML BM-MSCs and AML blast cells. The top 11 genes (>10+ fold change) that are differentially expressed between AML BM-MSCs and AML blasts observed were in the expression of collagen and extracellular matrix proteins (*P*-value=6.5 × 10^−9^ to 4 × 10^−8^).

BM-MSCs from AML patients (Figure 1a) and healthy donors (Figure 1b) expressed comparable amounts of β_1_ integrin (CD29), CD44, CD73, CD90 and E-cadherin (CD324). The normal BM stromal cell lines, HS-5 (Figure 1c) and HS-27a (Figure 1d), demonstrated surface marker expression similar to that of normal marrow MSCs. Confirming mesenchymal origin, BM-MSCs from AML patients expressed CD105 and CD146 (Figure 1a), and gene expression profiling demonstrated large differences in expression with statistically significant enrichment of collagen type 1 and other connective tissue and extracellular matrix genes in BM-MSCs as compared to AML blasts (Figure 2). Low levels of CD45 and CD34 were occasionally detected, either due to leukemia cell or monocyte (CD45 or CD34) or endothelial cell (CD34) contamination.

### Cytokine profile of BM-MSCs from AML patients and healthy donors

To identify physiologic differences between the BM-MSCs from AML patients vs healthy donors, we analyzed cytokine production from the conditioned media of the BM-MSCs in hypoxic conditions (5% oxygen) and found no statistically significant differences in the levels of eight of the nine cytokines when comparing AML BM-MSCs to normal BM-MSCs (Table 1a). One notable exception was MCP-1, which was significantly reduced in BM-MSCs from AML patients (*P*=0.04) as compared to normal BM-MSCs from healthy donors (approximatly five times lower). We observed a trend toward reduced GM-CSF and IL-6 levels produced by AML BM-MSCs compared with BM-MSCs from healthy donors (*P*>0.07). In hypoxic conditions as compared to normoxic conditions, however, there was a strong reduction in cytokine production in AML BM-MSCs with significantly lower levels of GM-CSF, stem cell factor and TNF-α (*P*<0.04), and a trend toward lower levels of MCP-1 (*P*=0.09; Table 1b). Tables 2a and b provide the clinical characteristics of the AML patients for which cytokine levels were measured from their corresponding MSCs when cultured in hypoxic 5% oxygen and normal 21% oxygen conditions, respectively.

### Gene expression profile of BM-MSCs from AML patients and normal healthy donors

**Figure 3:**
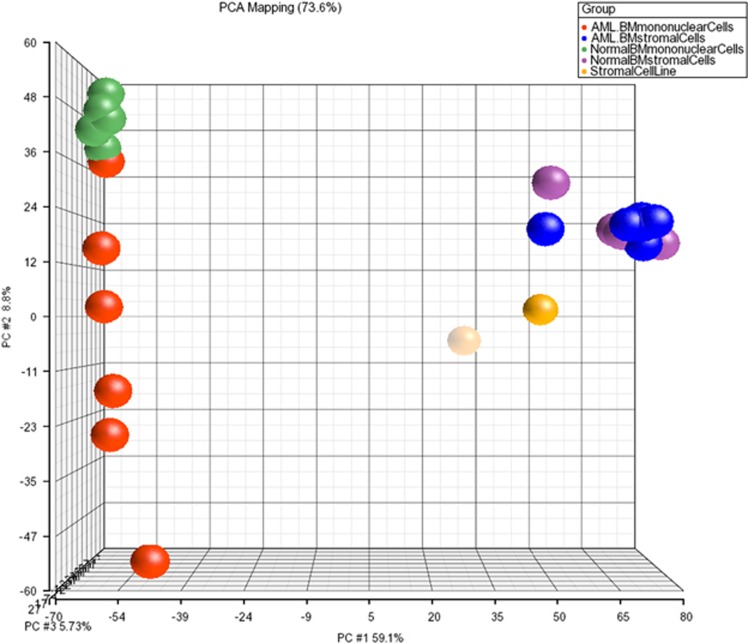
Principle component analysis generated from the microarray gene. Expression data identify differences between AML and normal BM mononuclear cells but no differences between AML and normal BM-MSCs. The plot represents 73% of the variance in the data. The first principle component (PC1) describes 59% of the variance in these data, the second principle component (PC2) describes 9% and the third component (PC3) describes 6%. Mononuclear cells (regardless from AML or from normal bone marrow) can be distinguished from the mesenchymal stromal cells mainly by PC1.

Table 2a provides the clinical characteristics of AML patients for which microarray analysis was performed. Principal component analysis (Figure 3) on the microarray data demonstrated no statistically significant differences in the gene expression profile of BM-MSCs from AML patients (blue spheres) compared to BM-MSCs from healthy donors (purple spheres). Gene expression within the normal or AML BM-MSC groups was homogenous, as was expression in the hematopoietic MNCs from healthy donors (green spheres). In contrast, the corresponding blasts from AML patients (red spheres) demonstrated significant individual variability in mRNA expression, in particular along the PC2 (principle component 2) axis. As expected, the mRNA (or gene) expression profile of BM-MSCs from AML patients demonstrated marked differences in gene expression compared to their corresponding AML blasts (Figure 2) in particular, as previously noted, high level expression of collagen and extracellular matrix components by BM-MSCs. These analyses demonstrate that (1) BM-MSCs from AML patients and healthy donors showed no significant difference in mRNA expression as measured by Illumina's BeadChip microarray platform and (2) AML blasts demonstrate increased inter-patient heterogeneity and have different mRNA expression profiles as compared to their corresponding BM-MSCs and normal hematopoietic MNCs. We then compared the differences between AML blasts and their MSCs vs normal hematopoietic MNCs and their stroma. Genes with the greatest difference in fold-change expression between AML blasts and AML BM-MSCs compared to normal hematopoietic MNCs and normal BM-MSCs are shown in Supplementary Table 2. The oncogenes FBJ murine osteosarcoma viral oncogene homolog (FOS) and v-myb avian myeloblastosis viral oncogene homolog (MYB) exhibit among the highest ratios of AML vs normal, 6.0 and 3.8, respectively, and the IPA network highlights these. Also notable are genes that are not part of the IPA network but are nevertheless important in the pathobiology of leukemia, including chemokine (C-X-C motif) receptor 4, CD96, colony stimulating factor 3 receptor and LIM domain only 2.

### Ingenuity pathway analysis

**Figure 4:**
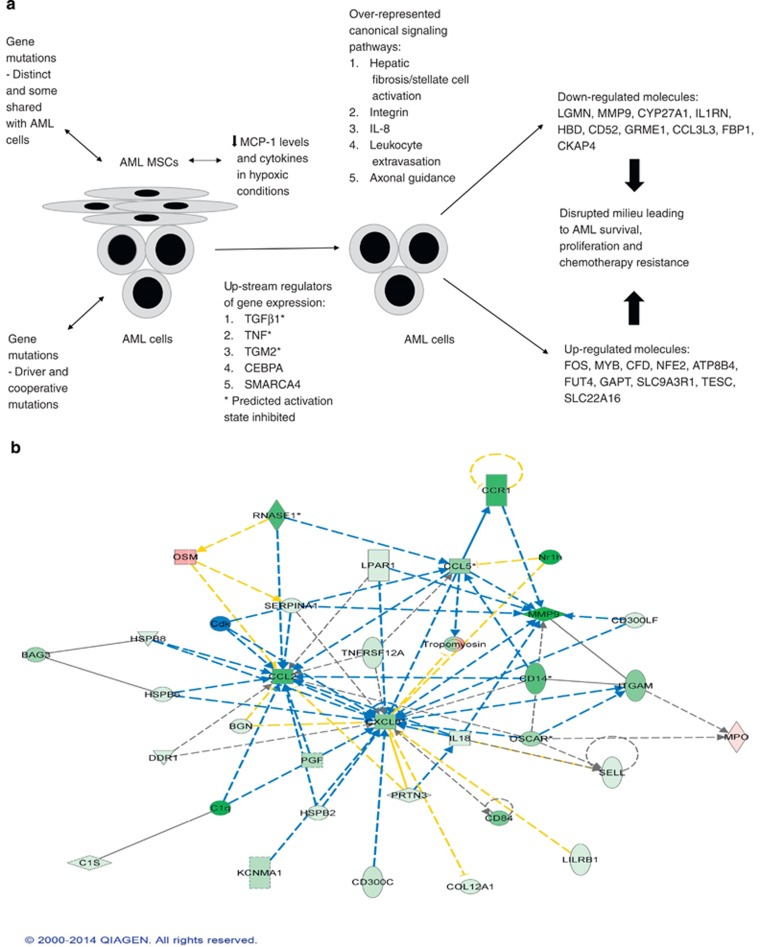
(**a**) Proposed model of dysfunctional bone marrow microenvironment in AML based on cytokine data and gene expression analysis through Ingenuity pathway analysis. (**b**) Ingenuity pathway analysis depicting the network of pathways that lead to downregulation and inhibition of MCP-1/CCL2 from AML BM-MSCs from the microarray data. Green nodes indicate downregulation. Red nodes indicate upregulation. Blue dotted lines indicate predicted indirect inhibition. Blue solid line indicates predicted direct inhibition. Gray solid lines indicate effect not predicted or no causal association. Gray dotted lines indicate no direct or indirect association between the molecules. Yellow solid lines indicate no clear pattern of activation or inhibition. Yellow dotted lines indicate no clear pattern of direct or indirect interaction (activation or inhibition) between the nodes.

IPA analysis was used to identify putative regulatory, functional or pathway-specific gene expression differences between AML BM-MSCs and AML blasts compared with the differences in gene expression between normal BM-MSCs and normal hematopoietic MNCs from healthy donors. The results of this analysis showed key distinctions in the leukemia-derived population to be in: (1) upstream regulation of gene expression by transforming growth factor beta 1 (TGFβ1), TNF, tissue transglutaminase 2 (TGM2), CCAAT/enhancer binding protein alpha (C/EBPA) and SWItch/Sucrose NonFermentable (SWI/SNF) related, matrix associated, actin dependent regulator of chromatin, subfamily a, member 4 (SMARCA4) (*P*-values 4.81 × 10^−21^ to 1.26 × 10^−38^), with the predicted activation states of TGFβ1, TNF, TGM2 inhibited; and (2) hepatic fibrosis/hepatic stellate cell activation, integrin, leukocyte extravasation, IL-8 and axonal guidance signaling as overrepresented canonical pathways (*P*-values 3.90 × 10^−13^ to 4.45 × 10^−17^). Figure 4a shows the results of the IPA in a proposed model of the dysfunctional BME in AML. Concordant with decreased MCP-1 in our cytokine profiling data, Figure 4b illustrates a gene expression network that contributes to inhibition of MCP-1 (chemokine (C-C motif) ligand 2 (CCL2)). See Supplementary Table 1 for description and function of the top 10 upregulated and downregulated molecules and Supplementary Figure 3 for representative pathway network showing upregulation of the transcription factors, FOS and MYB in AML.

### Cytogenetic analysis of BM-MSCs from AML patients

Given the cited murine model data showing how directed mutations of stromal cells can lead to myelodysplasia and emergence of AML,^12, 13^ we hypothesized that AML patients, at least in some cases, might demonstrate mutations in their BM-MSCs. We selected four AML patient samples with easily detectable cytogenetic aberrations present in their leukemic blasts that might allow comparison with BM-MSCs. Table 2c provides the clinical characteristics and conventional karyotype for the AML blasts and the BM-MSCs from these AML patients. In patient AML 070, the leukemic cells had a complex karyotype, while the AML BM-MSCs demonstrated a normal karyotype. For patient AML 098, the AML BM-MSCs demonstrated a distinct abnormal karyotype, while the leukemic cells demonstrated trisomy 14. In two patients, the AML BM-MSCs and leukemic cells demonstrated overlapping cytogenetic aberrations—*t(2;11)(q33;q13*) in patient AML 073 and *der(5;17)(p10;q10), −6, del(7)(q22q34), +8, −18, −20 and −22* in patient AML 094—as well as distinct aberrations. Thus, BM-MSCs in a small sample of AML patients demonstrate abnormal cytogenetics, some overlapping with and others distinct, from the cytogenetic aberrations present in their corresponding leukemic blasts.

## Discussion

AML arises from the clonal evolution of a hematopoietic progenitor cell that acquires successive genetic alterations. The mechanisms by which MSCs influence AML development and progression are under active investigation. We found significantly reduced MCP-1 levels from BM-MSCs from AML patients compared to BM-MSCs from healthy donors. MCP-1 is one of the major chemokines that regulate migration and infiltration of monocytes and macrophages. Apart from recruiting and directing leukocyte movement, MCP-1 also exhibits anti-tumor activity, in promoting Fas cell surface death receptor-ligand-mediated apoptosis in cultured endometrial stromal cells.^19^ MCP-1 is secreted by both AML blasts and stromal cells, and serum levels are increased in AML patients and appear to vary depending on AML subtype.^20^ Our data show that MCP-1, GM-CSF, stem cell factor and TNF-α levels produced by AML BM-MSCs are vastly different depending on oxygen levels and are much lower in hypoxic conditions similar to the sinusoidal regions of the BM. We observed a trend toward reduced GM-CSF and IL-6 production from AML BM-MSCs compared to normal BM-MSCs. The lower GM-CSF levels observed would be consistent with the view that the leukemic BME results in a diminished ability to support normal hematopoiesis.

We observed significant differences in gene expression in AML BM-MSCs compared to leukemic blasts, but not compared to BM-MSCs from healthy donors. IPA identified the regulatory pathways involving the genes that distinguished leukemia cells from their stroma (AML blasts vs AML MSCs) and those that distinguished normal hematopoietic cells from their stroma (normal hematopoietic MNCs vs normal MSCs) to be: (1) TGFβ1, TNF, TGM2, C/EBPA and SMARCA4 as upstream regulators of gene expression; (2) integrin and IL-8 signaling as overrepresented canonical pathways; and (3) upregulation of the transcription factors FOS and MYB. TGFβ1 has been shown to differentially regulate myeloid leukemia in the BME with suppressive effects on CML leukemia stem cells and proliferative effects on AML stem cells,^21^ and plasma-derived exosome content of TGFβ1 from AML blasts may predict AML relapse.^22^ TNFs have been shown to differentially affect cytokine expression and migration properties of MSCs.^23^ The pro-inflammatory effects of TNF increase AML survival and proliferation by upregulation of anti-apoptotic genes.^24^ Tissue transglutaminase 2 (TGM2) expression is elevated in AML along with many other cancer cell types and increased gene expression correlates with increased expression of adhesion and apoptosis-regulating proteins, such as fibronectin, integrin β3 and focal adhesion kinase.^25^ TGM2 expression is induced by TGFβ, TNF-α and IL-6, and evidence supports its common role in chronic inflammation and cancer.^26^ The *C/EBPA* gene belongs to the CCAAT/enhancer-binding protein family of transcription factors, which is a master regulator of hematopoietic cell proliferation and differentiation and is frequently mutated in AML. Double mutations in the *C/EBPA* gene confers a relatively favorable prognosis in AML.^27^ SMARCA4 is a catalytic subunit of the SWI/SNF (brahma related gene 1 (BRG1) associated factor) chromatin regulatory complex, which is frequently mutated in cancer and has recently been shown to be critical for AML maintenance and proliferation.^28, 29^ Integrin β3-mediated signaling has been shown to be required for leukemia development in a xenotransplantation study.^30^ IL-8 is a pro-angiogenic and chemotactic cytokine, whose levels are increased in AML cells when co-cultured with fibroblasts and stromal cells, which likely contributes to the increased vessel density in AML BM and thus important in leukemogenesis.^31^

The IPA revealed upregulation of the transcription factors FOS and MYB, which regulate cell proliferation, differentiation and transformation. FOS, one the upregulated molecules in AML, forms a complex that regulates TGF-β signaling. A downstream target of TGFβ1 is MCP-1. Thus, the diminished MCP-1 production from AML BM-MSCs may be the result of FOS-mediated inhibition of TGF-β signaling. The IPA reveals that leukemic cells (AML BM-MSCs and AML blasts) show significant differences in upstream gene regulation and canonical pathway signaling, which results in a complex interplay of upregulation and downregulation of signaling molecules compared to normal cells (normal BM-MSCs and hematopoietic MNCs), and highlights potentially the most important pathways and molecules to target as biomarkers or for combinatorial therapeutic manipulation.

A previous study showed a distinctive gene expression profile of MSCs of MDS and AML patients compared with MSCs of healthy donors using microarray analysis.^32^ In contrast to the present study, we did not identify any significant differences gene expression in the BM-MSCs from AML patients compared to the BM-MSCs of healthy donors. This discrepancy could in part be explained by the age difference in the study population, with the previous study involving pediatric cases of MDS/AML. The BME undergoes changes with aging, such as increased adipogenesis and decreased osteoblastogenesis, and an aged BM may facilitate better expansion of leukemic cells compared to a young BM.^33^ In addition, the length of time in culture, number of passages and tissue culture media could explain the differences in findings. A study by Jacamo *et al.*^34^ recently showed that primary BM-MSCs from leukemia patients expressed nuclear factor kappa beta target genes at higher levels than their normal BM-MSC, both upon co-culture with leukemic cells and in monoculture. In their study, the BM-MSCs were in culture for 4 to 8 weeks before genetic analysis. We did not detect differences in our AML BM-MSCs compared to normal BM-MSCs from healthy donor in gene expression profiling in monoculture at early passages.

A study by Flores-Figueroa E *et al.*^35^ showed no significant functional differences between MDS-derived BM-MSCs and normal BM-MSCs, with similar expression of cell adhesion and extracellular matrix proteins, ability to differentiate and support hematopoiesis *in vitro*, even among stromal cells with severe chromosomal abnormalities. Another group, however, showed decreased MSC plasticity and hematopoietic supportive capacity in higher-risk MDS patients,^36^ suggesting that there may be heterogeneity. In support of previous studies, we also observed that BM-MSCs from AML patients expressed similar cell-surface markers and adhesion proteins compared to BM-MSCs from healthy donors, such as β1 integrin, CD44, CD73, CD90, and E-cadherin.

We found BM-MSCs from AML patients demonstrated cytogenetic aberrations either distinct from or overlapping with their corresponding leukemic blasts. Several groups have demonstrated cytogenetic aberrations in BM-MSCs from MDS patients,^15, 35, 37, 38^ and one group has described aberrations in BM-MSCs in adult AML^15, 39^ that were distinct from the leukemic cells. To the best of our knowledge, our study represents the first report of AML BM-MSCs sharing some similar chromosomal aberrations with their corresponding leukemic blasts. Whether chromosomal aberrations lead to functional alterations in BM-MSCs and how this influences disease progression and outcome in leukemia patients remain to be fully elucidated. In the report by Blau *et al.*,^15^ the presence of chromosomal aberrations in BM-MSCs was associated with inferior overall survival and disease-free survival. However, on multivariate analysis, cytogenetic changes in BM-MSCs were not an independent prognostic factor when the hematopoietic cell karyotype was included as a variable. We did not evaluate the relationship of BM-MSC aberrations to prognosis in our study due to the small sample size.

In summary, we found reduced MCP-1 levels from primary BM-MSCs from AML patients compared to normal BM-MSCs from healthy donors and that cytokine secretion from AML BM-MSCs vary depending upon oxygen conditions, with much lower levels in hypoxic conditions that approximate the sinusoidal regions of the BME. The surface antigen and gene expression profile did not differ between BM-MSCs from AML patients compared to normal BM-MSCs. We found a few cases in which BM-MSCs from AML patients had overlapping and distinct cytogenetic aberrations with their corresponding leukemic cells. It will be of interest to evaluate the effect of manipulating MCP-1 levels in AML in future studies and to further characterize BM-MSCs with abnormal cytogenetics and the influence of reduced cytokine production in a hypoxic BME on leukemia stem cell quiescence.

## Figures and Tables

**Table 1:** Cytokine levels (mean, pg/ml) measured from primary BM-MSCs in AML patients in comparison with normal healthy donors

	*G-CSF*	*GM-CSF*	*IL12p70*	*IL-6*	*MCP-1*	*M-CSF*	*SCF*	*TNF-α*	*SDF-1β*
*a*									
Normal stroma, 5% O_2_	0	16.17	0	18 747	1699	10.52	0	1.16	0
AML stroma, 5% O_2_	0	0	0	11 394	327	2.35	0	1.2	0
									
*b*									
AML stroma, 21% O_2_	0.82	30.69	4.35	23 490	51 640	4.75	6376	27.2	1134
AML stroma, 5% O_2_	0	0	0	11 394	327	2.35	0	1.2	0

Abbreviations: AML, acute myeloid leukemia; BM-MSCs, bone marrow-derived mesenchymal stromal cells; G-CSF, granulocyte colony-stimulating factor; GM-CSF, granulocyte-monocyte CSF; IL, interleukin; M-CSF, macrophage CSF; MCP-1, monocyte chemoattractant protein-1; SCF, stem cell factor; SDF-1β, stromal-derived factor-1β TNF-α, tumor necrosis factor-α.

(a) When cultured in hypoxic (5% O_2_) conditions, AML BM-MSCs showed no statistically significant differences in the levels of most cytokines tested including G-CSF, IL-12, IL-6, M-CSF, SCF, TNF-α and SDF-1β. AML BM-MSCs showed reduced MCP-1 (*P*-value=0.04) and GM-CSF (*P*-value=0.07) levels compared to normal BM-MSCs. (b) When AML BM-MSCs from a group of patients are cultured in hypoxic (5% O_2_) conditions (*n*=6) and compared to an independent cohort (except for AML 034, *n*=5) cultured in normal (21% O_2_) oxygen conditions, a significant decrease in GM-CSF, SCF and TNF-α levels was observed (*P*<0.04) in hypoxic conditions. There was a trend toward decreased MCP-1 levels (*P*=0.09) in hypoxic conditions.

**Table 2:** Characteristics of AML patients

*Patient ID*	*Age at diagnosis (years)*	*Gender*	*Diagnosis*	*Cytogenetic data (AML blasts)*
*a*				
AML 034	57	F	AML with MDS-related features	Normal
AML 038	51	F	AML M1	Normal
AML 039	51	M	AML M4	Normal
AML 070	30	F	AML M5	46,XY,t(5;8;15)(q31;q24.3;q11.2), t(6;13)(p23;q12),t(10;15)(q22;q15)[7]/46,XY,t(3;5)(p21;q22),del(15)(q11.2q15)[7]/46,XY[6]
AML 073	62	M	AML M4	46,XY,t(2;11)(q33;q13),t(6;9)(p23;q24)[20]
AML 075	69	M	AML with MDS-related features	44,XY,del(3)(p21),der(5)t(5;11)(q11.1;q11),-11, 12,del(16)(q12.1),der(16;?)(p13.3;?),-17,add(20)(q12),+mar1[18]/46,XY[2]
				
*b*				
AML 017	63	F	Relapsed M4Eo	46,XX,inv(16)(p13q22)[14]/46,XX,add(7)(q32),inv(16)(p13q22)[5]/47,XX,inv(16)(p13q22), +22[2].nuc.ish(CBFBx2)(5'CBFB sep 3'CBFBx1)[132/200]
AML 029	26	M	M4	46,XY,inv(16)(p13.1q22)[18]/47,sl,+mar1[2]
AML 030	54	F	Relapsed M5	Normal
AML 032	30	M	Therapy-related AML	t(8;21), del9q
AML 034	57	F	AML with MDS-related features	Normal

*Patient ID*	*Age at diagnosis (years)*	*Gender*	*Diagnosis*	*Cytogenetics (AML blasts)*	*Cytogenetics (AML BM-MSCs)*
*c*					
AML 070	30	F	Relapsed AML M5	46,XY,t(5;8;15)(q31;q24.3;q11.2), t(6;13)(p23;q12),t(10;15)(q22;q15)[7]/ 46,XY,t(3;5)(p21;q22),del(15)(q11.2q15)[7]/46,XY[6]	Normal 46,XY [23]
AML 073	62	M	AML M4		46,XY,t(2;11)(q33;q13), del(4)(q12q21)[23]
AML 094	48	M	AML with MDS-related features	45~47,X,add(Y)(q11.23),add(4)(q12),der(5;17)(p10;q10),-6,del(7)(q22q34),+8,-18,add(18)(q11.2),-20,-22,+1~3r,+mar1,+2~4mar[cp20]	47,X,-Y,−4,der(5;17)(p10;q10),−6,del(7) (q22q34),+8,+16,-18,-20,-22,+6mar[1]/46,XY[19].nuc ish(D8Z2x3)[5/600]
AML 098	48	M	AML M4	47,XY,+14[20]	46,XY,add(5)(q13)[5]/46,XY[24]

Abbreviations: AML, acute myeloid leukemia; F, female; M, male; MDS, myelodysplastic syndrome.

(a) Clinical and cytogenetic characteristics of AML patients (*n*=6) used for gene expression analysis and cytokine production assay in hypoxic conditions (5% O_2_). (b) Clinical and cytogenetic characteristics of AML patients (*n*=5) used for cytokine production assay in normal O_2_ conditions (21%). (c) Clinical and cytogenetic characteristics of AML patients (*n*=4) used for detection of cytogenetic aberrations in their corresponding BM-MSCs.
