# Supplementary material for: Mesenchymal stromal cells derived from acute myeloid leukemia bone marrow exhibit aberrant cytogenetics and cytokine elaboration
Source: Blood Cancer J. 2015 Apr 10;5(4):e302–. doi: 10.1038/bcj.2015.17 (PMC4450324; doi:10.1038/bcj.2015.17)
Supplement: Supplementary Information [file bcj201517x1.docx]

**Supplementary Figure 1**. **(A)** Hierarchical clustering was generated for genes

differentially expressed when comparing AML blast cells to AML BM-MSCs with

normal BM-MSCs and normal hematopoietic mononuclear cells. Color intensity is

scaled within each row so that the highest expression value corresponds to bright red

and the lowest to bright green. Gene symbols are listed on the right. **(B)**

Representative close-up sections from the global heat map. The first 6 columns

correspond to individual AML patients leukemic blast samples. The next 6 columns

correspond to individual AML patients BM-MSCs. The next 5 columns correspond to

individual normal healthy donor bone marrow hematopoietic mononuclear cells. The

last 5 columns correspond to individual normal healthy donor BM-MSCs. Gene symbols

are listed on the right. There are significant differences in gene expression between

AML blasts compared to normal hematopoietic mononuclear cells while AML BM-MSCs

and normal BM-MSCs are similar in their gene expression.

**Supplementary Figure 2.** Spindle-shaped morphology of bone-marrow derived mesenchymal stromal cells from AML patients, normal healthy donor and a normal stromal cell line (HS-5).

**Supplementary Figure 3** - Representative IPA network depicting the pathways that

lead to up-regulation of the transcription factors FOS and MYB in AML. Green nodes

indicate down-regulation. Red nodes indicate up-regulation. Orange nodes indicate

predicted activation. Blue nodes indicated predicted inhibition. Blue dotted lines indicate

predicted indirect inhibition. Blue solid lines indicate predicted direct inhibition. Orange

dotted lines indicate predicted indirect activation. Orange solid lines indicate predicted

direct activation. Gray lines indicate effect not predicted. Yellow lines indicate findings

inconsistent with state of downstream molecule.

**Supplementary Table 1**. Top 10 up-regulated and down-regulated molecules from Ingenuity Pathway Analysis comparing the differences in gene expression between leukemia-derived cells (AML MSCs and blasts) with the differences in gene expression between normal cells (normal MSCs and hematopoietic mononuclear cells).

A description of the up and down-regulated molecules and their functions are provided:

Up-regulated molecules

1. FOS (FBJ murine osteosarcoma viral oncogene homolog) - Interacts with JUN

family of proteins to form the transcription factor complex AP-1, which regulates

cell proliferation, differentiation and transformation.

1. CFD (complement factor D) - Alternative complement pathway protein. Also

secreted by adipocytes.

1. MYB (V-Myb avian myeloblastosis viral oncogene homolog) – Transcription

factor involved in regulation of the control of proliferation and differentiation in

hematopoietic progenitor cells.

1. NFE2 (nuclear factor, erythroid 2) – Regulates erythroid and megakaryocytic

maturation and differentiation.

1. ATP8B4 (ATPase, class 1, type 8B, member 4) – involved in phospholipid

transport in cell membrane.

1. FUT4 (fucosyltransferase 4) – Catalyzes synthesis of the non-sialylated antigen,

Lewis x (CD15) and associated with myeloid sarcoma.

1. GAPT (GRB2-binding adaptor protein, transmembrane) – Negative regulator of

B-cell proliferation.

1. SLC9A3R1 (solute carrier family 9, subfamily A, member 3 regulator 1) –

Sodium/hydrogen exchanger regulatory cofactor that regulates beta2-adrenergic

receptor and parathyroid hormone 1 receptor. Links integral membrane and

cytoskeletal proteins.

1. TESC (tescalin) – cofactor in regulation of cellular pH through sodium/hydrogen

exchange; induces hematopoietic stem cell differentiation toward megakaryocytic

and granulocytic lineages.

1. SLC22A16 (solute carrier family 22, member 16) – transports carnitine and other

organic cations.

Down-regulated molecules:

1. LGMN (legumain) – Regulates cell proliferation through degradation of internalized epidermal growth factor receptor.
2. MMP9 (matrix metallopeptidase 9) – Involved in extracellular matrix degradation,

specifically type IV and V collagens; may be involved in IL-8 induced mobilization

of hematopoietic progenitor cells from bone marrow.

1. CYP27A1 (cytochrome P450, family 27, subfamily A, polypeptide 1) – Involved in

drug metabolism and cholesterol biosynthesis.

1. IL1RN (interleukin 1 receptor antagonist) – Inhibits IL-1, IL1A and IL1B and

modulates immune and inflammatory responses.

1. HBD (hemoglobin, delta) – involved in oxygen transport.
2. CD52 - may play role in carbohydrate orientation.
3. GREM1 (gremlin 1) – bone morphogenic protein antagonist member; inhibitor of

monocyte chemotaxis.

1. CCL3L3 (chemokine ligand 3-like 3) – chemotaxis of lymphocytes and

monocytes.

1. FBP1 (fructose-1,6-bisphosphatase 1) – regulator of gluconeogenesis.
2. CKAP4 (cytoskeleton-associated protein 4) – anchors endoplasmic reticulum to

microtubules.

**Supplementary Table 2** – Genes with the greatest fold change in expression between AML cells vs. AML mesenchymal stromal cells and normal hematopoietic mononuclear cells vs. normal mesenchymal stromal cells are shown (ratio of AML fold change to normal fold change >2 or <0.5).
