# Supplementary material for: Mesenchymal stromal cells derived from acute myeloid leukemia bone marrow exhibit aberrant cytogenetics and cytokine elaboration
Source: Blood Cancer J. 2015 Apr 10;5(4):e302–. doi: 10.1038/bcj.2015.17 (PMC4450324; doi:10.1038/bcj.2015.17)

**Supplemental Information, Figure 1**. Global heat map **(A)** and representative close-up sections **(B)** of the heat map of differentially expressed genes comparing AML blasts to normal hematopoietic mononuclear cells to AML BM-MSCs to normal BM-MSCs. The labels for the columns are identical for A and B, and legible in B. The first set of columns represent the AML blasts, the second set normal bone marrow mononuclear (NBM) cells, the third AML derived mesenchymal stromal cells, and the fourth normal bone marrow (NBM) derived mesenchymal stromal cells. Note the similarities between AML and NBM mesenchymal stromal cells, with the distinct differences between AML gene expression and normal BM mononuclear cells, and between the hematopoietic cells vs. the stromal cells.

**A.**


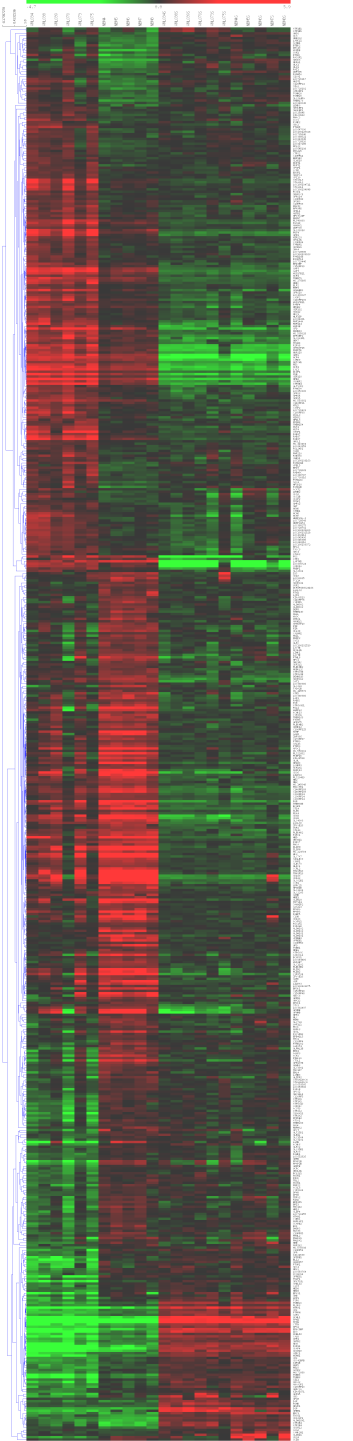


B.


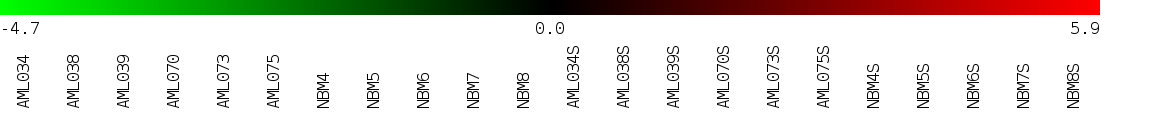

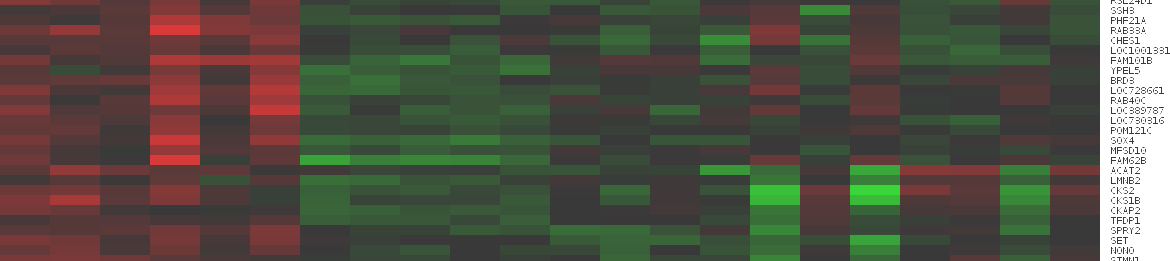

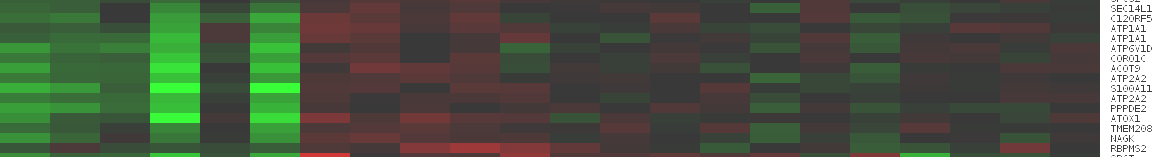


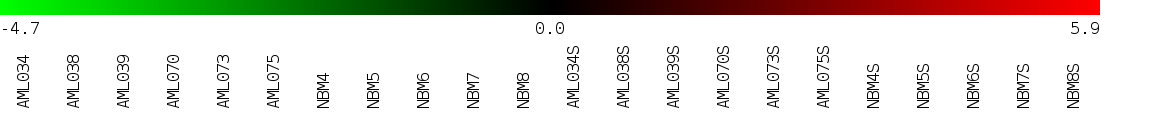

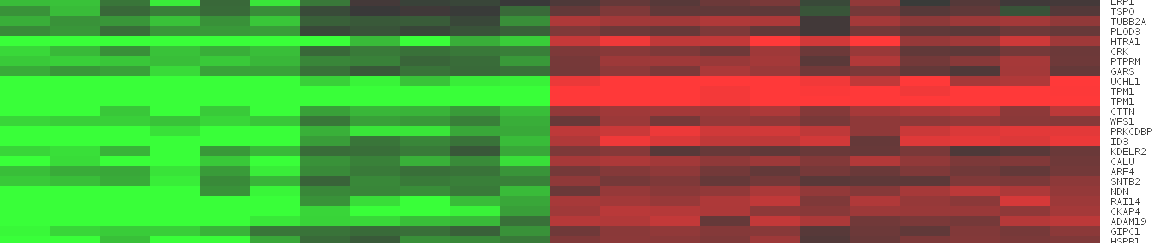

Supplement: Supplementary Figure 1 [file bcj201517x2.docx]
