# Supplementary material for: Mesenchymal stromal cells derived from acute myeloid leukemia bone marrow exhibit aberrant cytogenetics and cytokine elaboration
Source: Blood Cancer J. 2015 Apr 10;5(4):e302–. doi: 10.1038/bcj.2015.17 (PMC4450324; doi:10.1038/bcj.2015.17)
Supplement: Supplementary Figure 2 [file bcj201517x3.docx]

**Supplementary Figure 2.** Spindle-shaped morphology of bone-marrow derived mesenchymal stromal cells from AML patients, normal healthy donor and a stromal cell line (HS-5).


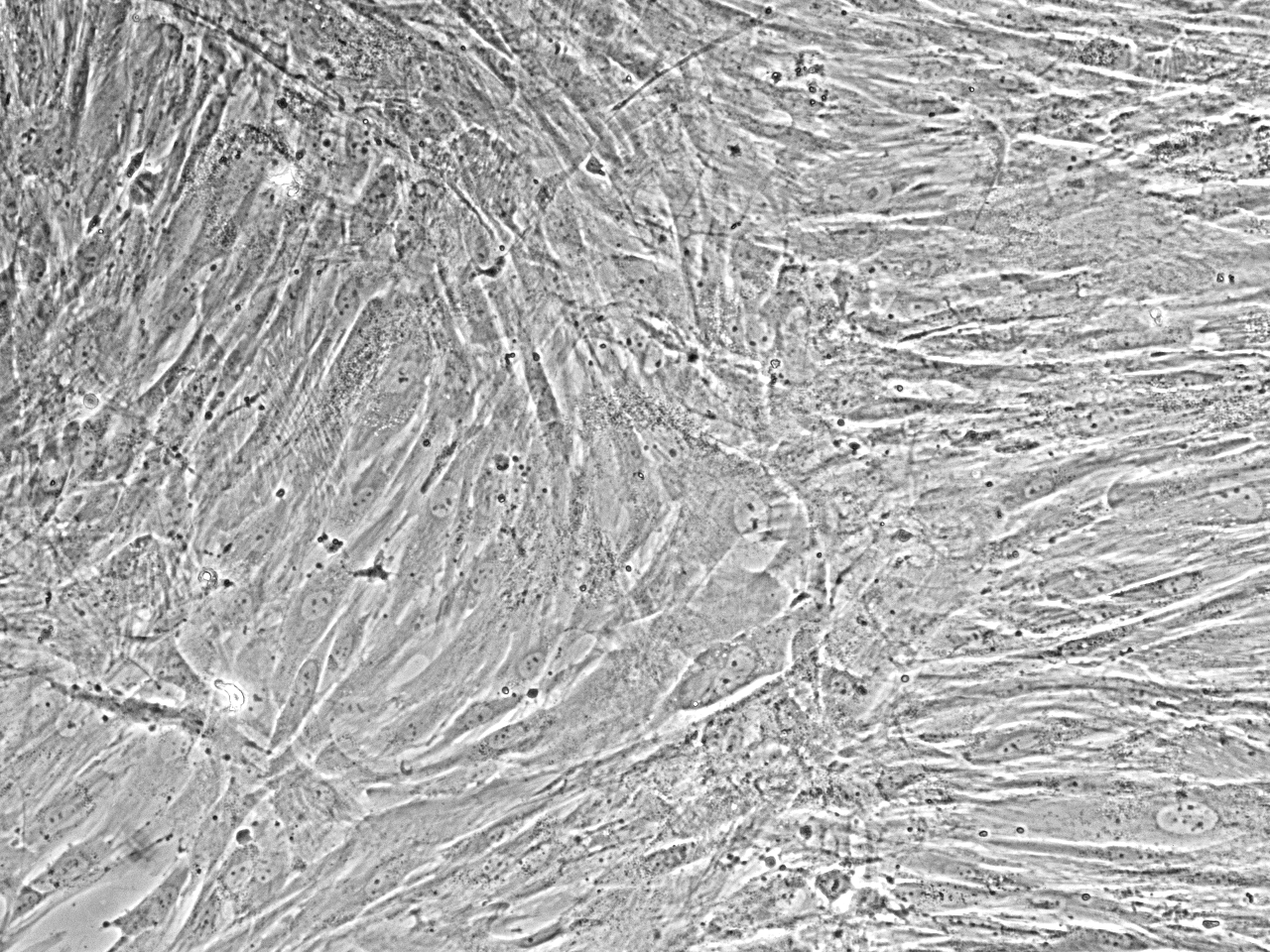

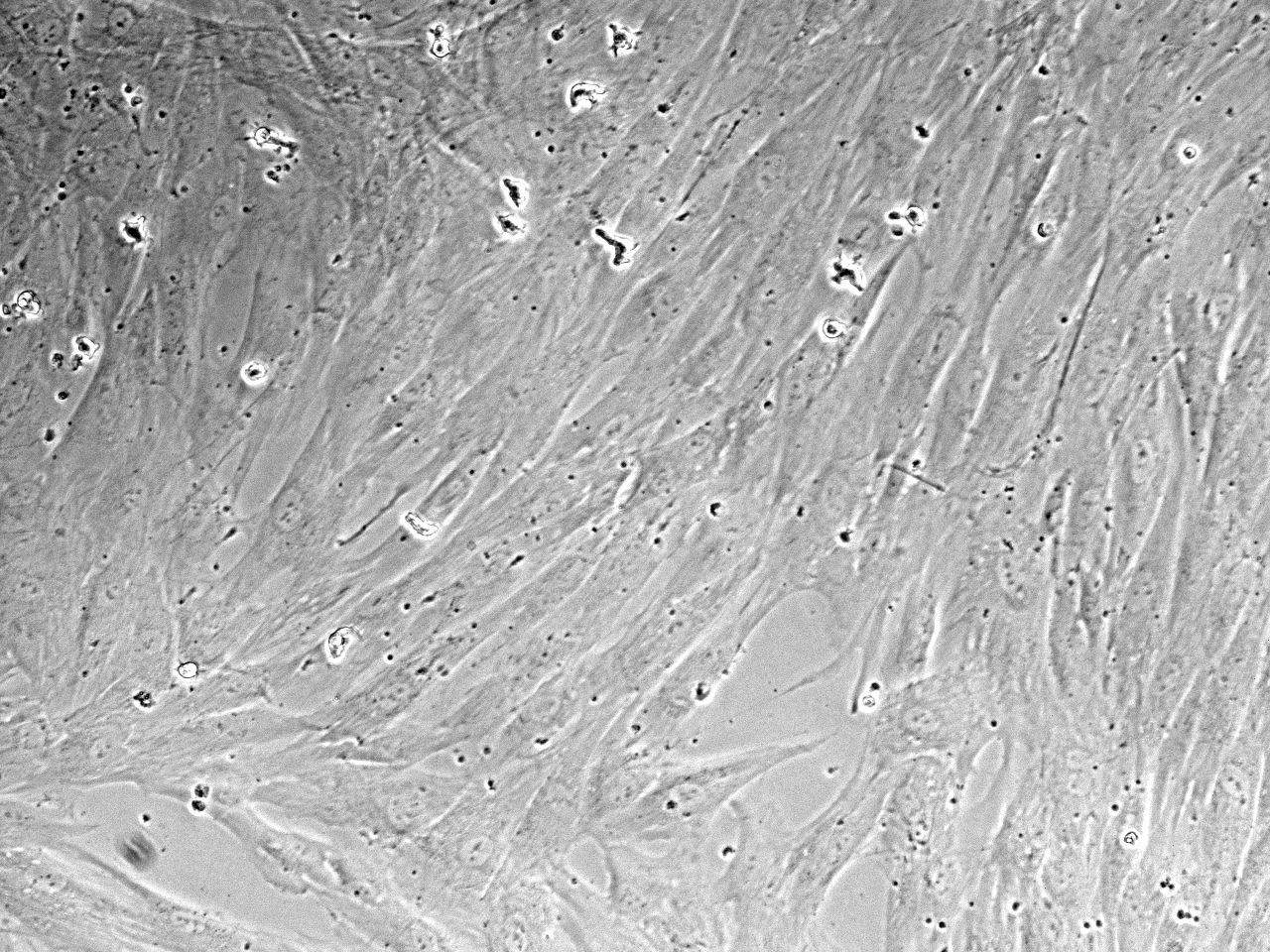

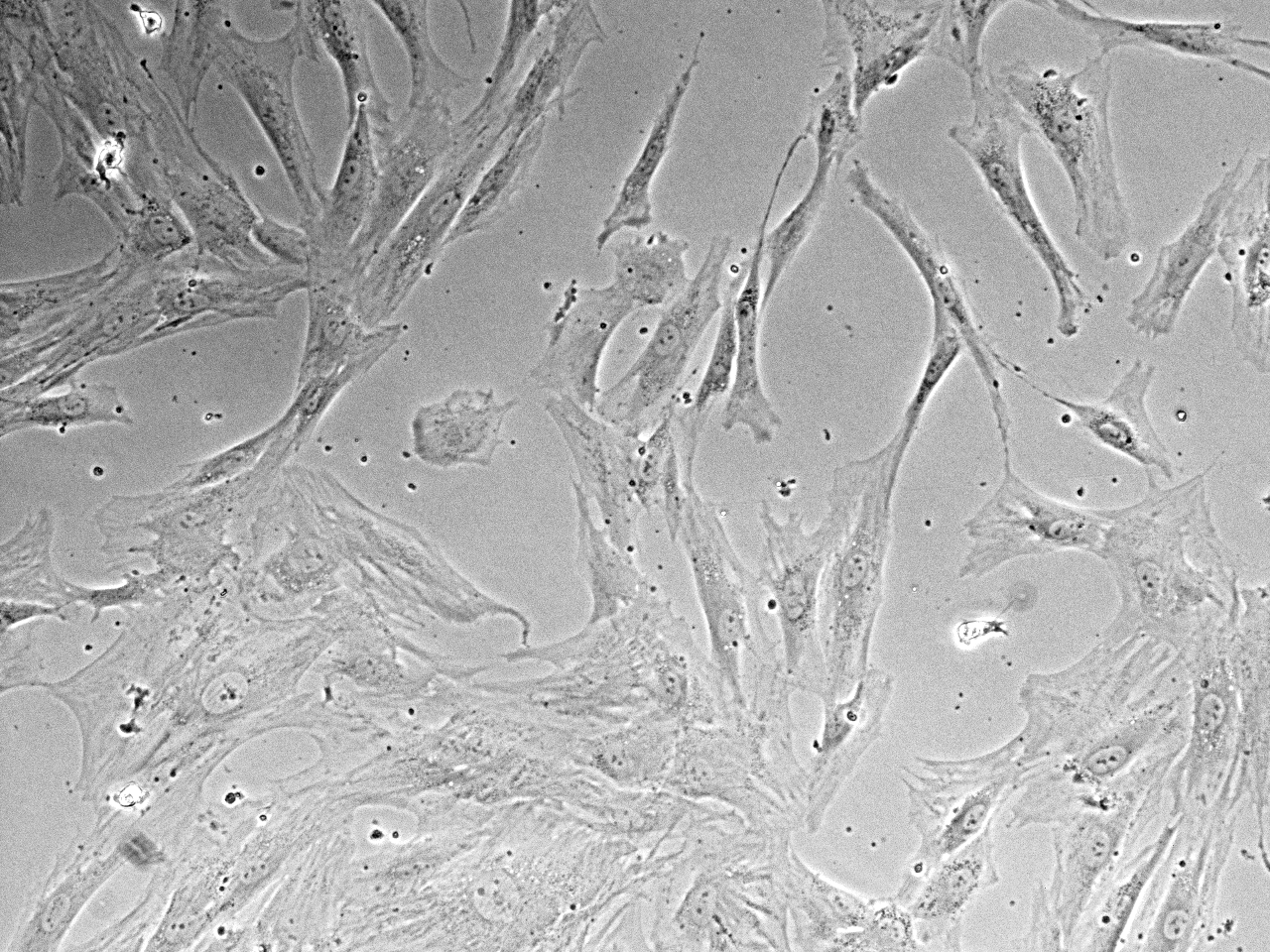

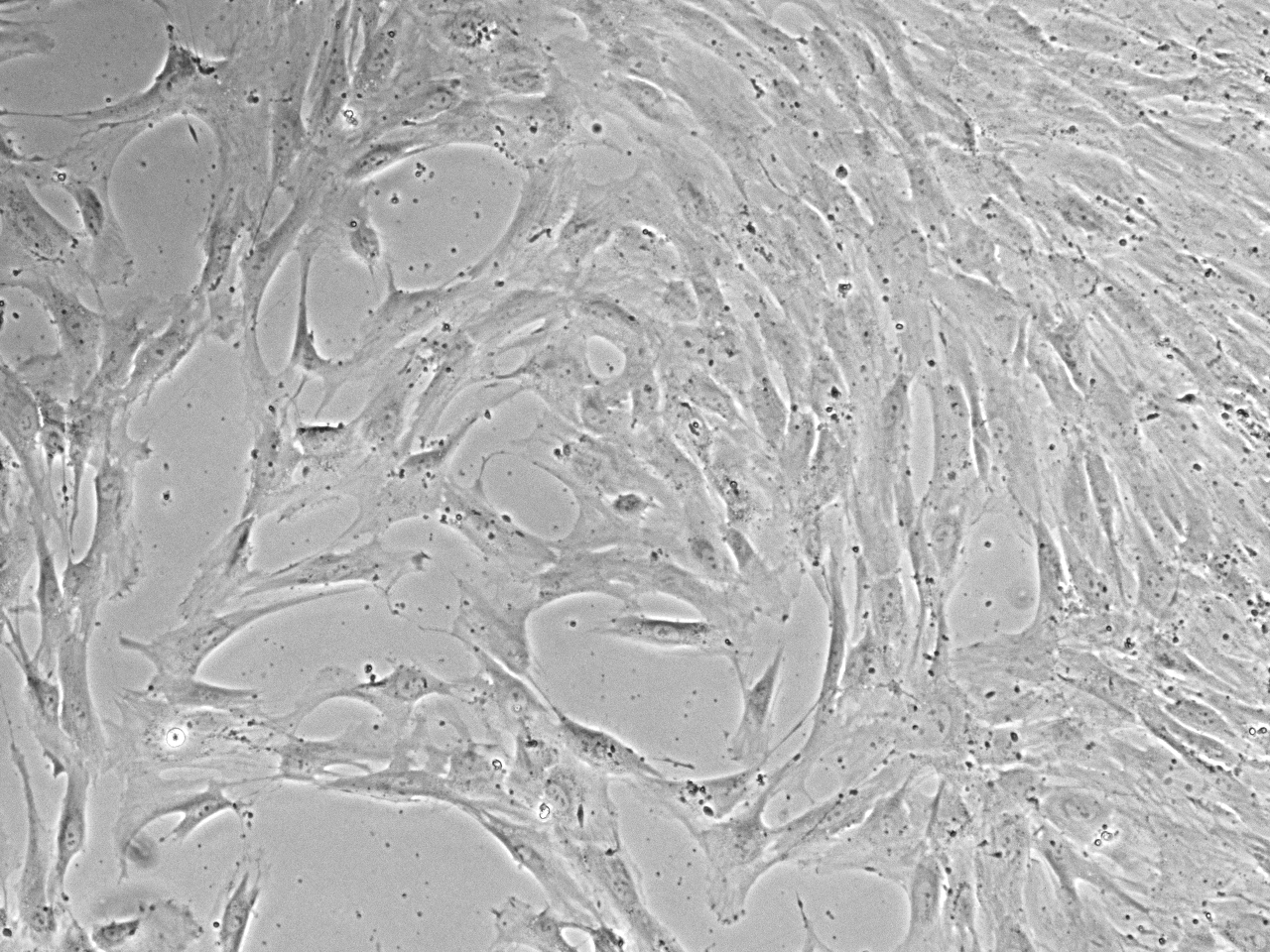


**Primary AML Stromal Cells**


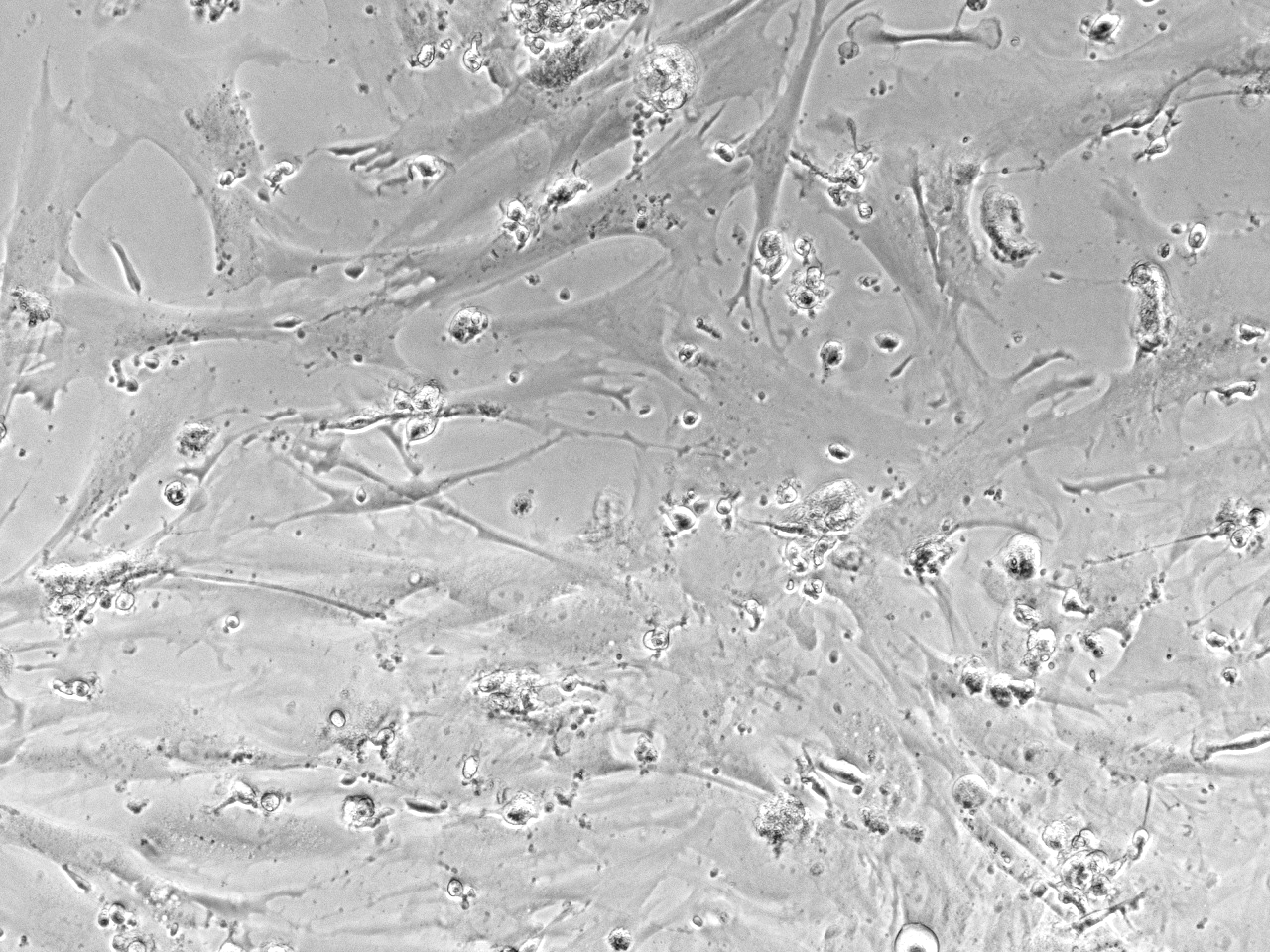


**Primary Normal BM Stromal Cells**


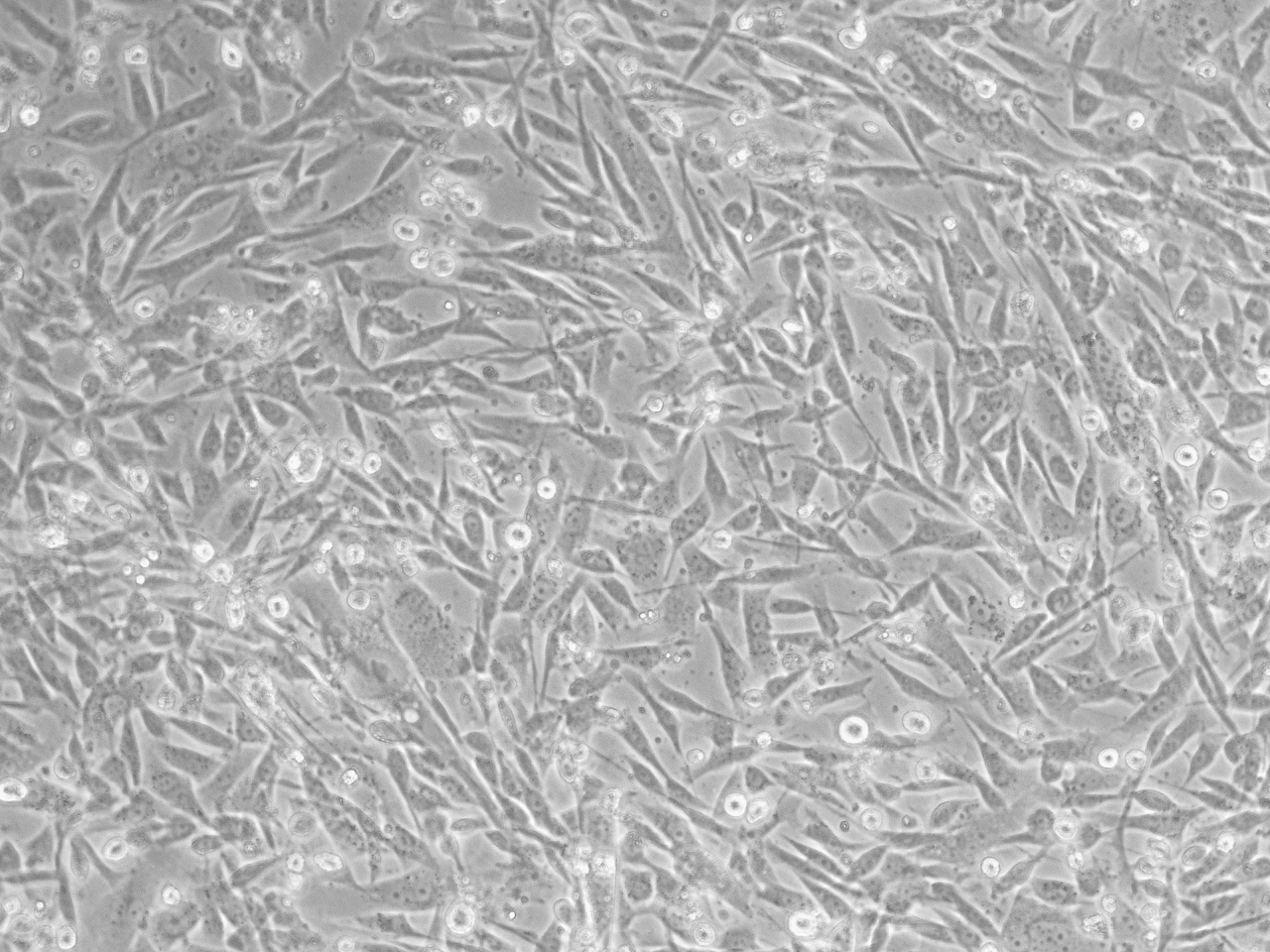


**Stromal Cell Line HS-5**
